# Real-Time Determination of Molecular Weight: Use of MaDDOSY (Mass Determination Diffusion Ordered Spectroscopy) to Monitor the Progress of Polymerization Reactions

**DOI:** 10.1021/acspolymersau.4c00020

**Published:** 2024-05-09

**Authors:** Owen Tooley, William Pointer, Rowan Radmall, Mia Hall, Thomas Swift, James Town, Cansu Aydogan, Tanja Junkers, Paul Wilson, Daniel Lester, David Haddleton

**Affiliations:** † Department of Chemistry, University of Warwick, Coventry CV4 7AL, United Kingdom; ‡ Department of Chemistry, University of Bradford, Bradford BD7 1DP, West Yorkshire, United Kingdom; § School of Chemistry, Monash University, 17 Rainforest Walk, Clayton, VIC 3800, Australia; ∥ Polymer Characterization RTP, University of Warwick, Coventry CV4 7AL, United Kingdom

**Keywords:** diffusion ordered spectroscopy, NMR, polymerization, reaction monitoring, mass determination, online monitoring

## Abstract

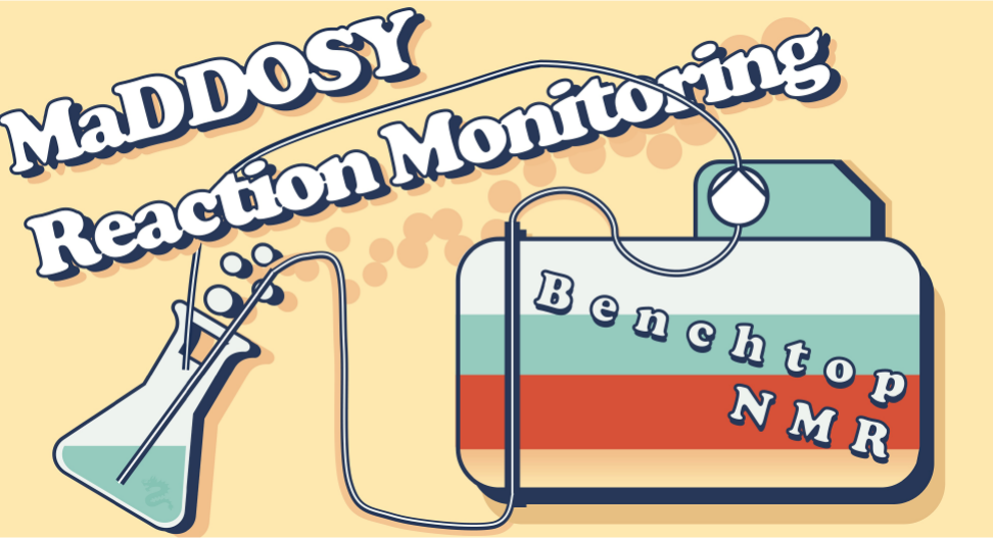

Knowledge of molecular weight is an integral factor in polymer synthesis, and while many synthetic strategies have been developed to help control this, determination of the final molecular weight is often only measured at the end of the reaction. Herein, we provide a technique for the online determination of polymer molecular weight using a universal, solvent-independent diffusion ordered spectroscopy (DOSY) calibration and evidence its use in a variety of polymerization reactions.

## Introduction

The molecular weight and dispersity of a polymer largely determine the physical properties of a material. As such, measurement of molecular weight information both of the final product and how it evolves during the reaction is important. However, in most cases, the techniques required for this type of characterization require offline analysis. The modern standard benchmark technique for this in a synthetic laboratory is gel permeation chromatography (GPC) or, in some cases, mass spectrometry.^1^ These techniques provide useful information on both molecular weight and dispersity; however, they generally require sample preparation or workup from the reaction mixture before analysis, rendering these techniques particularly challenging for reaction monitoring in real time and any subsequent automative reaction optimization.

This notwithstanding, there have been notable successes in this area; the exemplary review by Haven and Junkers^2^ gives an excellent overview of these achievements. The first analyses of polymerizations acquired through mass spectrometry were performed by Santos et al. in 2008^3^ in which the Brookhart polymerization of alkenes was monitored using electrospray ionization (ESI) mass spectrometry. In that work, the authors coupled the reaction vessel directly to the ESI source, in conjunction with inline dilution with acetonitrile before the mass spectrometer. This required no workup, with rapid analysis times of just a few seconds for each measurement. However, this approach is limited to ionizable polymers and those that produce only low-molecular weight species and is also complicated by multiple charges on any individual polymer, making a complex system even more complex. These are inherent problems pertinent to real-time reaction monitoring. As most high-molecular-weight polymers present difficulties for analysis by ESI due to the combination of multiple charge states, mass discrimination along with the distribution of chemical specials produces a very large number of peaks, leading to complex analysis.^4^

Matrix-assisted laser desorption ionization (MALDI) can be used, which typically has only singly charged species, but this often requires laborious sample preparation, and automation is not trivial. Several matrices are often required to be screened to ensure acceptable ablation and ionization from the MALDI plate, but several orders of magnitude of dilution, combination with a salt or other ionizing agent, and precise spotting on a plate are required to collect data of a desirable quality. MALDI also favours lower mass molecules as both ionization and detection are mass sensitive favouring lower masses. Due to this discrimination against higher masses, the weight average molecular weight is always underestimated which leads to dispersity being significantly underestimated. There have, however, been several reported instances where MALDI has been used to characterize polymerization reactions: Wu et al. used MALDI to study the mechanism of the ring-opening polymerization (ROP) of tetrahydrofuran (THF) using bis(pentafluorophenyl)(phenoxy)borane,^5^ and Scherger et al. used the technique to study self-immolative RAFT-polymer end-group modification.^6^ However, in both of these cases, sample preparation was performed manually, with analysis being subsequently performed. Automated MALDI analysis has shown some promise since its inception by Meier et al., where a home-built MALDI spotter was integrated with a high-performance liquid chromatography (HPLC) system and applied to study polymer end groups.^7^ Its use has become more widespread, evidenced by Pirrone et al., who used it for the rapid screening of poly(ethylene glycol) conjugated cytokines.^8^ Indeed, there are now commercially available MALDI spotters capable of integration with existing systems^9^ to reduce preparation and analysis time.

Clearly, there still exists a need for online polymer molecular weight analysis in real time. GPC provides many benefits. First, sample preparation is simple, often only requiring dissolution to an appropriate concentration and then filtering.^10^ GPC can be readily used for polymers in excess, from very low *M*_wt_ to >1,000,000 g mol^–1^, and is applicable in a wide range of solvents, allowing for analysis of a significantly wide range of polymers. There are, however, some innate drawbacks of GPC; first is that even the best GPC systems are generally accepted to possess an error of up to 10%,^11^ largely arising from the use of standards that do not typically match the chemical characteristics of the analyzed polymers. Results from GPC are solvent-dependent, as the hydrodynamic volume, the parameter measured in GPC, can be affected by how well the solvent solvates the polymer.^12^ Thus, problems can arise, for example, some polymers, such as PET and nylon, are only soluble in very particular solvents, such as fluorinated acids, or require elevated temperatures, complicating analysis.^13^ Other polymers, particularly those that are water-soluble, can cause problems due to interacting with the column, meaning that retention is a result of not only size but also chemical interactions, vastly changing the retention time and therefore the calculated molecular weight. Further complication arises due to GPC being a chromatographic method, and hence, each individual evaluation can take between 5 and 45 min, severely limiting the available time resolution in online analysis and the quality of the result if the analysis time is pushed down.

This notwithstanding, there have been several instances of success of applying GPC for online reaction monitoring. As early as 2004, work was being focused on the online monitoring of controlled radical polymerizations by GPC detector methods, and this is well-exemplified in the work by Mignard et al.,^14^ in which the authors used light scattering (LS), viscometry, differential refractive index (DRI), and UV detectors to characterize gradient copolymerization reactions. In this work, excellent temporal resolution was achieved, and the groundwork for truly online characterization of polymerization reactions has been described. In this instance, molecular weight was calculated using concentration and light-scattering data, requiring a significant amount of information to be known about the system of interest prior to reaction monitoring being carried out, somewhat limiting universal applicability to new systems of interest. In 2010, Levere et al.^15,16^ combined RI and LS detectors using a rapid GPC column to allow for the monitoring of single-electron transfer living radical polymerization (SET-LRP) in close to real time, with a temporal resolution of approximately 4 min. Due to the use of rapid GPC columns, only poor chromatographic resolution was obtained, and in order to obtain higher resolution, more conventional GPC columns are required, often with run times >30 min, which limits the reactions to be monitored to those >3 h in order for meaningful data to be obtained. More recent developments by both the Junkers and Warren groups^17,18^ using online GPC techniques have produced exciting results. In both of these cases, the relative ease of data analysis from online GPC has been used to build self-optimization algorithms for the high-throughput testing of polymerization conditions, thereby reducing the need for multiple, time-consuming batch reactions. This work shows that there is clearly scope and interest for automated online analyses of molecular weight for a multitude of reasons. Indeed, in both of the cases referenced, GPC was used in conjunction with other analysis techniques, notably NMR, to measure conversion.

This use of NMR in automated, online polymer synthesis is increasing due to the availability of benchtop NMR systems, which give increasingly good resolution, allowing NMR to be a much more accessible technique in the synthetic laboratory. NMR has been used to monitor the progress of many polymerizations, usually by performing reactions in a standard NMR tube often within the magnet of a high-field cryogenic instrument^19,20^ or sampling from a reaction at given time points and subsequently performing the analysis offline. Full online reaction monitoring within a typical laboratory scale in real time has been prohibitive. The reasons for this are plentiful, including the usual requirement for deuterated solvents, the need for specialized flow cells, the need for liquid cryogens for magnet cooling, and the lack of laboratory infrastructure in close proximity to NMR spectrometers. Benchtop NMR systems address the majority of these problems and have been used for 1D NMR experiments for a range of polymerizations.^21,22^ First, as most employ an external lock to prevent magnetic field drift,^23^ deuterated solvents are not required. This provides a significant benefit for reaction monitoring, as experiments can be performed at a standard laboratory scale in conventional solvents, which are often far less expensive than their deuterated counterparts. Benchtop NMR systems require no cryogenic cooling, which vastly reduces the physical space required for the magnet, thus allowing them to be transported around a laboratory on trolleys, making them inherently portable. This much smaller size also means that flow cells can be much smaller and generally composed of standard glass (or plastic) tubing with conventional HPLC flow fittings. These factors combined mean that the NMR spectrometer can be brought into the synthetic laboratory, rather than the other way around. Thus, reactions can be performed under a multitude of conditions, including under an inert atmosphere, at variable temperature, and at scale. The reaction mixture can then be pumped directly through the NMR spectrometer and returned without significant deviation from the required conditions.

A further recent development in benchtop NMR spectroscopy is the advances and application of gradient-based solvent suppression. A particularly useful experiment in polymer science is the diffusion ordered spectroscopy (DOSY) experiment. The DOSY experiment is becoming increasingly used in polymer science. It shows particular use in the assessment of the success of copolymerization reactions and as a tool to demonstrate an increase in size at the conclusion of a reaction.^24^

The measured diffusion constant can be correlated with the molecular weight of the macromolecule, and indeed, this has been shown in previous works^25,26^ and further developed into a universal calibration within our groups.^27^

There do exist some limitations of this approach, detailed in our previous work. Briefly, the solvent must act as a “good” solvent for the system of interest, meaning that it should readily solubilize the polymer. Additionally, the concentration must be carefully controlled, based on the expected final molecular weight of the polymer, to a concentration known as C*; for polymers in the molecular weight range of 1000–100,000 g mol^–1^, this is approximately 20–50 mg mL^–1^.^28,29^ While this does not provide an excessive number of problems for reaction monitoring, a slightly more dilute system than typically used for batch polymerizations is required. Additionally, knowledge of the approximate molecular weight of the final polymer is required, meaning that reasonably well-understood chemistries are needed. A further limitation is the requirement to know the bulk viscosity of the solvent of interest. For most common laboratory solvents, this value is known at ambient temperature and can be easily found in literature tables;^30^ however, values at elevated temperatures are more of a challenge to find. As such, for some reactions, this value must be recorded experimentally first, either through traditional techniques such as Ostwald viscometry or via the use of an inline viscometer.

Once the viscosity and concentrations are controlled, however, the approach can, in principle, be used to monitor reactions in real time. Indeed, the use of DOSY to monitor reactions of small molecules in flow has been reported by Marchand et al.^31^ and its use for polymerizations has been reported by Vrijsen et al.^32^ In both cases, high-field NMR instruments were used, and in the instance of the polymerization reaction, no universal calibration was used to determine molecular weight, limiting application to a wider range of polymers and polymerizations.

The use of DOSY with a benchtop NMR system, and our previously reported universal calibration, therefore presents an interesting opportunity as a possible tool for the determination of molecular weight in real time for the monitoring of a wide range of polymerizations of different monomers under different reaction conditions.

## Experimental Section

### Materials and Instrumentation

Methyl acrylate, 1,4-dioxane, azobis(isobutyronitrile) (AIBN), ascorbic acid, *tert*-butyl hydroperoxide, ethyly-2-bromoisobutyrate (EBiB), CuBr_2_, cyclohexane, tetrahydrofuran, and isoprene were purchased from Merck. The RAFT agent, 2-(((butylthio)carbonothioyl)thio)propanoic acid (PABTC), and tris[2-(dimethylamino)ethyl]amine (Me_6_Tren) were synthesized according to previously reported literature.^33,34^

All NMR spectra were acquired on a Magritek Spinsolve 80 Carbon Benchtop NMR spectrometer equipped with a *z*-axis gradient coil capable of generating a maximum field gradient of 500 mT/m. 1D ^1^H NMR spectra were interpreted using Magritek Spinsolve versions 2.1.3 or 2.3.6. DOSY spectra were interpreted using Magritek Spinsolve version 2.3.6 or the GNAT, version 1.3.2, developed by the Manchester NMR Methodology Group.^35^ GNAT was used for the anionic and RAFT experiments. Spinsolve was used for the Cu-RDRP experiment.

GPC samples were recorded using an Agilent Infinity II MDS instrument equipped with differential refractive index (DRI), viscometry (VS), dual angle light scatter (LS), and multiple wavelength UV detectors. The system was equipped with 2× PLgel Mixed C columns (300 × 7.5 mm^2^) and a PLgel 5 μm guard column. The eluent was either CHCl_3_, in the case of polyisoprene, or THF with 0.01% BHT additive, in the case of poly(methyl acrylate). Samples were run at 1 mL/min at 30 °C. Poly(methyl methacrylate) and polystyrene standards (Agilent EasiVials) were used for calibration. Ethanol was added as a flow rate marker. Analyte samples were filtered through a nylon membrane with 0.22 μm pore size before injection. Experimental molar mass (*M*_n_, *M*_w_, SEC) and dispersity (*Đ*) values of synthesized polymers were determined by conventional calibration using Agilent GPC/SEC software.

### Polymerizations

**Figure 1:**
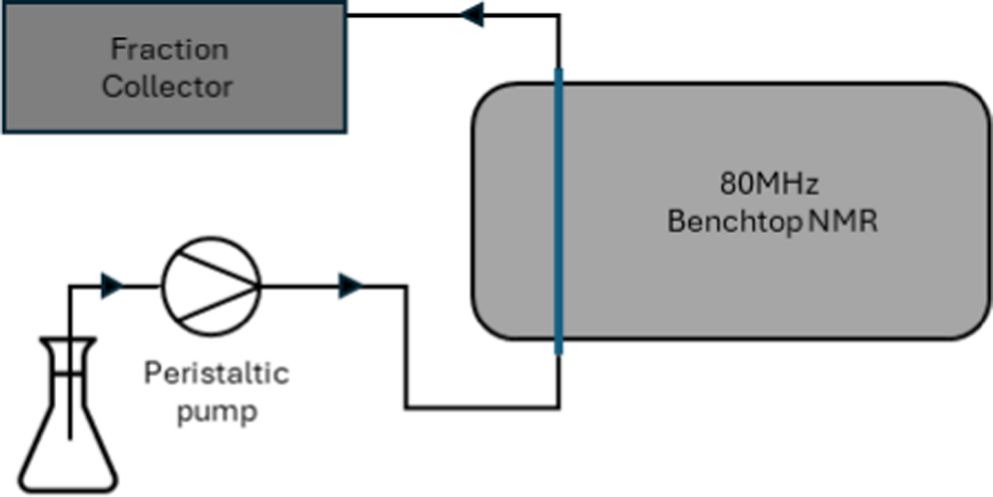
Polymerization reaction setup 1.

In the cases of anionic and RAFT polymerizations, the setup used was as shown in Figure 1. Briefly, this comprises a reaction vessel, from which the reaction mixture is pumped out into a glass flow cell/tube within the NMR spectrometer for measurement, after which it is pumped to a fraction collector, which either collects the sample or pumps the solution to waste. 1D ^1^H spectra, DOSY spectra, and fractions were collected every 90 min. All instrumental control was performed through an automated script, which is available in the Supporting Information. In each case, the 1D ^1^H spectra acquisition parameters were as follows: number of scans = 4, acquisition time = 6.4 s, repetition time = 15 s, and pulse angle = 90°. The DOSY parameters, used in the PGSTE pulse sequence, were optimized for the approximate target molecular weight of each system. This involved the following: number of scans = 4 or 8 (selected to provide a good signal from the polymer peak), number of gradient steps = 8, acquisition time = 6.4 s, repetition time = 15 s, and maximum gradient, big delta, and little delta were optimized to provide good attenuation of the polymer peaks through the gradient steps.

**Figure 2:**
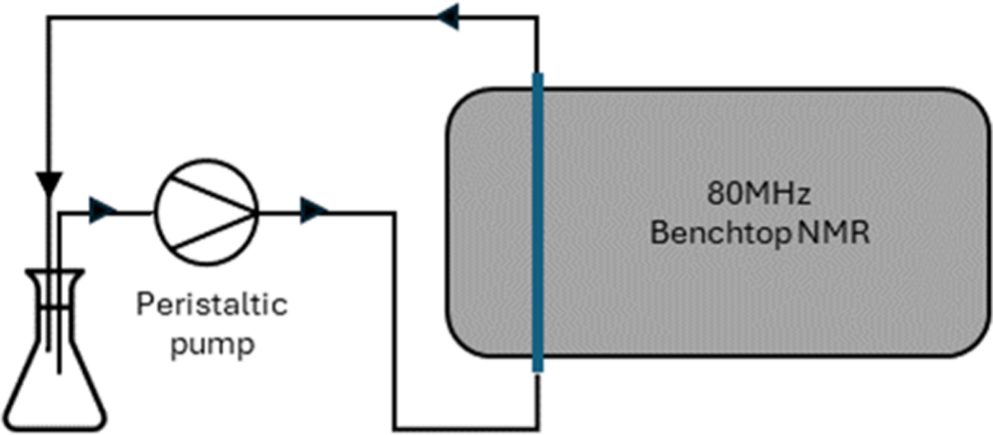
Polymerization reaction setup 2.

In the case of the Cu-RDRP reaction, the setup outlined in Figure 2 was used. This comprises a similar setup as in Figure 1, with the omission of the fraction collector and subsequent recirculation of the analyzed mixture back into the reaction vessel.

Molecular weights were calculated using the calibration detailed in our previous work,^36^ using the freely available associated online web-tool. For reference, the MaDDOSY calibration equation is shown in eq 1.
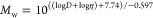
1

### Redox-Initiated RAFT Polymerization of Methyl Acrylate

Methyl acrylate (28.5 g, 0.33 mol) and RAFT agent (1.58 g, 6.6 mmol) were dissolved in 1,4-dioxane (270 mL) to give an approximately 10 wt % solution. The resultant solution was deoxygenated, brought under nitrogen, and left to equilibrate for 20 min at ambient temperature. The flask was then prepared for reaction monitoring according to Figure 1, and the solution was pumped through the system for priming. The automated script was started, and after the first system shim was performed, *tert*-butyl hydroperoxide (148.7 mg, 1.65 mmol) and ascorbic acid (145.3 g, 0.825 mmol) were added. The reaction was allowed to proceed for 720 min at ambient temperature, with spectra and fractions collected as previously described. After the reaction, the resultant fractions were reduced to a yellow viscous oil via gentle warming with a heat gun and subsequently dissolved in THF (1 mL) for GPC analysis. Conversion (^1^H NMR) 70%, *M*_w_ (GPC) 2800 g mol^–1^, *M*_wt_ (MaDDOSY) 3600 g mol^–1^, *Đ* (GPC) = 1.11

### Thermally Initiated RAFT Polymerization of Methyl Acrylate

Methyl acrylate (28.5 g, 0.33 mol) and RAFT agent (789 mg, 3.3 mmol) were dissolved in 1,4-dioxane (270 mL) to give an approximately 10 wt % solution. The resultant solution was deoxygenated, brought under nitrogen, and heated to 70 °C in an oil bath. The flask was then set up for reaction monitoring according to Figure 1, and the solution was pumped through to prime the system. The automated script was started, and after the first system shim was performed, AIBN (54.2 mg, 0.33 mmol) was added. The reaction was allowed to proceed for 810 min at 70 °C, with spectra and fractions collected as previously described. After the reaction, the resultant fractions were then reduced to a yellow viscous oil prior to dissolution in THF (1 mL) for GPC analysis. Conversion (^1^H NMR) 90%, *M*_w_ (SEC) 7500 g mol^–1^, *M*_wt_ (MaDDOSY) 7400 g mol^–1^, *Đ* (GPC) = 1.36

### Anionic Polymerization of Isoprene

Isoprene (20.43 g, 0.30 mol) was dissolved in cyclohexane (270 mL) to give an approximately 10 wt % solution, both of which had been dried over molecular sieves (3 Å), in a flask under nitrogen to give moisture contents of 8.3 ppm for cyclohexane and 10.7 ppm for isoprene as measured by Karl Fischer titration. The flask was placed in a reflux setup under nitrogen and left to equilibrate for 20 min at ambient temperature. Reaction monitoring was as above. Each collection vessel in the fraction collector had an aliquot of methanol (0.5 mL) in order to quench the reaction following fraction collection. The automated script was started, and after the first system shim was performed, *n*-butyl lithium solution in hexanes (2.1 mL, 1.4 M) was injected through a rubber septum. The reaction was allowed to proceed for 870 min at ambient temperature, with spectra and fractions collected as previously described. After the reaction, the resultant fractions were then reduced to a colorless, viscous oil with heating and subsequently dissolved in chloroform (1 mL) for GPC analysis. Conversion (NMR) 90%, *M*_w_ (GPC) 15,400 g mol^–1^, *M*_wt_ (MaDDOSY) 15,200 g mol^–1^, *Đ* (GPC) = 1.43

### Photoinitiated Cu-RDRP Polymerization of Methyl Acrylate

Methyl acrylate (20 mL, 0.222 mol), EBiB (326 μL, 2.22 mmol), CuBr_2_ (9.9 mg, 44.4 μmol), Me_6_TREN (71 μL, 0.226 mmol), and DMSO (180 mL), to give an approximately 10 wt % solution, were added to a round-bottom flask that was sealed with a septum and deoxygenated with nitrogen for 15 min. The flask was then set up for reaction monitoring according to Figure 2 and prepared as above. The automated script was started, and after the first system shim was performed, polymerization was started once the reaction mixture was placed inside a custom-made UV box with λ_max_ ∼ 360 nm. The reaction was allowed to proceed for 630 min at room temperature, with spectra collected as previously described. Conversion (^1^H NMR) 93%, *M*_w_ (GPC) 10,400 g mol^–1^, *M*_wt_ (MaDDOSY) 13,800 g mol^–1^, *Đ* (GPC) = 1.18

## Results and Discussion

### Reaction Monitoring Setup

In order to accurately measure the diffusion constant in a DOSY experiment, a time period, Δ, is required. This is commonly referred to as “big delta” and is a period of time in which diffusion is allowed to freely occur, after which the initial applied gradient is reversed prior to the measurement. The gradient itself can be applied in any Cartesian axis; however, this is often dictated by the hardware in each individual spectrometer. In the case of the spectrometer used in this work (Magritek), the gradient must be applied in the *z*-axis, which, unfortunately, is the direction in which the flow cell of the magnet resides. The result of this is that for the time period Δ*t*, the flow must be stopped to allow natural diffusion to take place and to allow the diffusion constant to be accurately measured. This results in a stop-flow setup, whereby for the duration of the DOSY measurement, the flow through the flow cell is stopped and then subsequently restarted once the measurement is complete. For the reactions in this work, this presents no issues; however, for implementation in a full-flow chemistry setup, it is noted that a switching valve would be needed to divert the flow during the stopped measurement phase, which has been shown as a possibility on a high-field spectrometer.^32^

### Viscosity Measurements

For the MaDDOSY calibration to be as accurate as possible, the viscosity of the system must be known. In the cases of dioxane in the redox-initiated RAFT polymerization, the cyclohexane in the anionic polymerization, and the DMSO in the Cu-RDRP polymerization, literature values were used.^37−39^ For the dioxane in the thermally initiated RAFT polymerization, temperature literature data were not available due to the increased temperature and the viscosity was therefore determined experimentally using Ostwald’s viscometry and was found to be 0.607 mPa·s.

### Redox-Initiated RAFT Polymerization of Methyl Acrylate

To first assess the feasibility of using the MaDDOSY system to monitor a polymerization reaction in real time, the first polymerization mechanism chosen was redox RAFT. This was chosen as the reaction could be carried out at ambient temperature while allowing for the potential of good control of dispersity and, being an RDRP process, should show linear evolution of molecular weight with respect to monomer conversion.^40^

**Figure 3:**
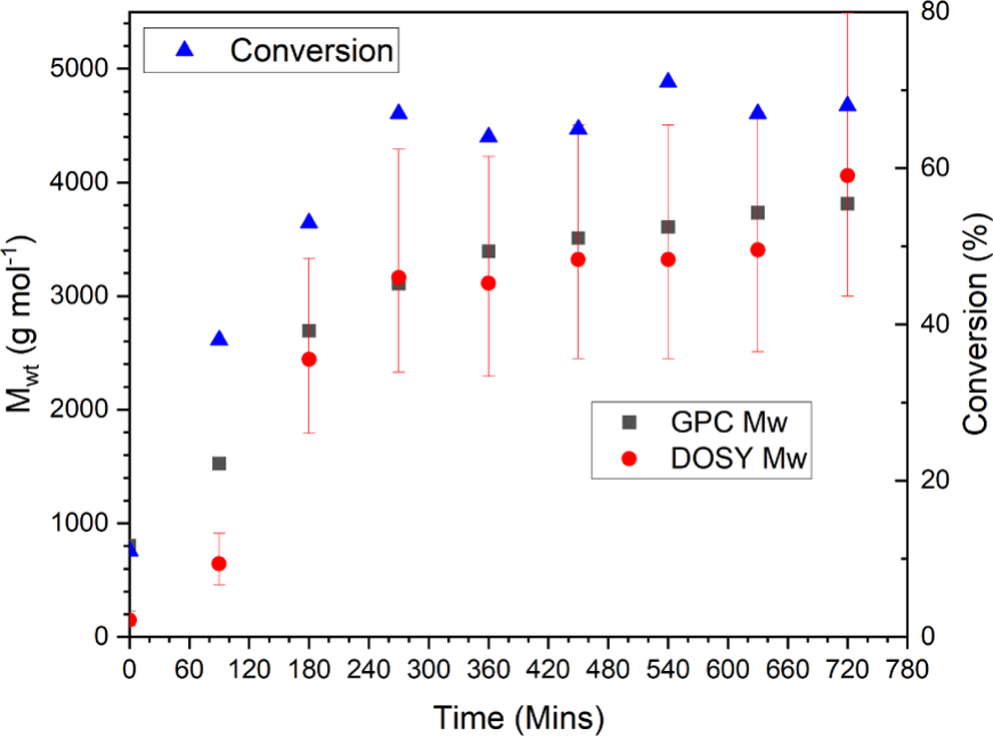
*M*_wt_ vs time for redox-initiated RAFT polymerization of methyl acrylate.

The molecular weight and monomer conversion with respect to reaction time are shown in Figure 3, with molecular weights calculated online via MaDDOSY, and, for comparison, the molecular weights from conventional GPC analysis are also plotted.

There is excellent agreement between the values obtained from GPC and from the MaDDOSY real-time monitoring. The uncertainties given in the MaDDOSY results are 95% confidence intervals, calculated as described previously.^36^ While these uncertainties seem large, they can be greatly reduced with the use of more scans in the NMR experiment; however, this of course comes at the cost of sampling frequency. It should also be noted that GPC results are often very close to the measured value from the MaDDOSY experiment, and as such, the 95% confidence intervals are very conservative in their approximation. The GPC results were obtained using narrow polystyrene and poly(methyl methacrylate) standards using conventional GPC analysis making use of a differential refractive index detector. There is less good agreement between the two techniques at the lower molecular weights seen early in the reaction likely due to two two factors. First, the MaDDOSY calibration is not as robust at molecular weights <1000 g mol^–1^. This is due to lower-molecular-weight polymers having less of a tendency to form ideal coils in solution due to their short chain length. Consequentially, the assumptions used in the MaDDOSY*—*that in a theta-like solvent the polymer forms idealistic chains and therefore the hydrodynamic radius is predictable*—*do not apply, and therefore, the predicted molecular weight is inaccurate. Second, the above arguments are also true for GPC, where lower molecular weight standards are often not available. However, once the molecular weights are >1000 g mol^–1^ and within the MaDDOSY calibration range, the agreement between the two techniques seems to be excellent.

**Figure 4:**
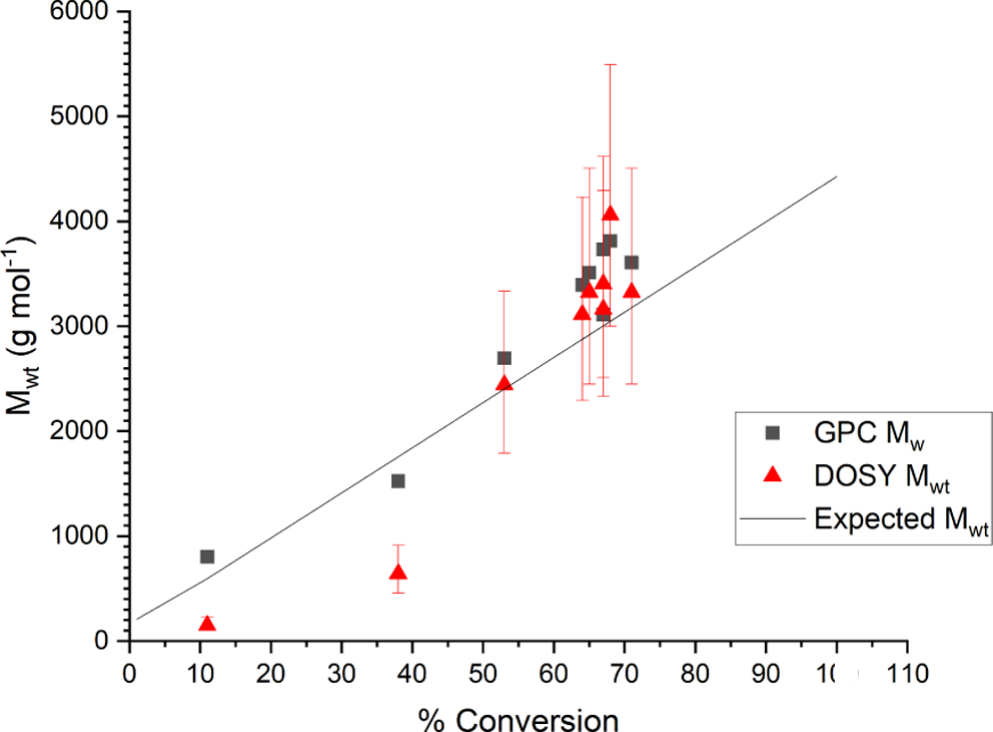
*M*_wt_ vs conversion for redox-initiated RAFT polymerization of methyl acrylate.

For this reaction, we would expect a linear growth of molecular weight with conversion. This is consistent with living (anionic) and RDRP processes,^41^ and this is indeed what was observed in Figure 4.

In these plots, *M*_w_ is plotted as the result from the GPC experiment; this is to allow comparison with the DOSY *M*_wt_, which most closely approximates *M*_w_. In the conversion plots, theoretical *M*_wt_ therefore assumes monodispersity. While a generally linear increase in *M*_wt_ with respect to monomer conversion is seen throughout the reaction, the reaction stops at approximately 70% conversion. This is a documented drawback of redox RAFT, which we do not fully understand,^42^ and it is noted that this does present challenges in terms of assessing the applicability of the reaction monitoring setup used; therefore, additional reaction mechanisms are required.

### Thermally Initiated RAFT Polymerization of Methyl Acrylate

**Figure 5:**
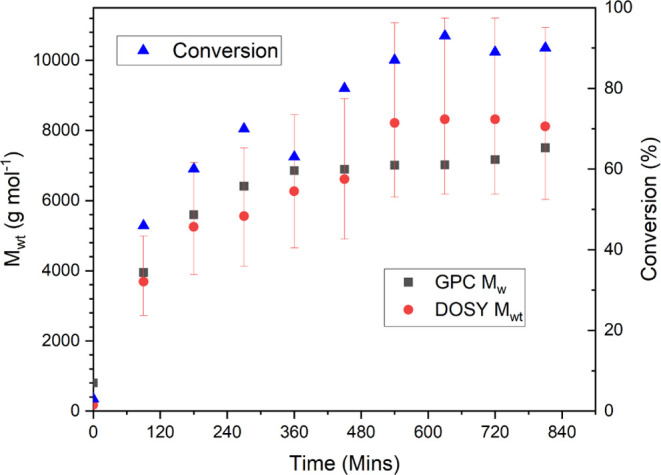
*M*_wt_ vs time for thermally initiated RAFT polymerization of methyl acrylate.

Probably the most common method of conducting a RAFT reaction is through thermal radical initiation, such as with azo initiators such as AIBN. As it is important to deliver radicals at a sufficient rate for the reaction to proceed, e.g., radical initiators with a 10 h half-life of between 50 and 120 °C,^43^ these elevated temperatures can cause problems for reaction monitoring. Diffusion is highly temperature-dependent, eq 1, as is viscosity. This results in the higher temperatures used in the MaDDOSY calibration no longer holding true, and as such, the calibration should be used with caution. The effects of this can be somewhat mitigated due to the viscosity correction, provided the viscosity at the given temperature is known and convection correction is accounted for.^26^ For the reaction studied here, conducted at 70 °C, the viscosity of dioxane was determined experimentally. This viscosity of course may not be the true viscosity for the DOSY measurement, as some degree of cooling will have occurred in the tubing; however, it remains a good approximation. Further work is aiming to create a temperature-corrected calibration, and its use would be more appropriate in the future.^26^ The *M*_wt_ increased linearly as a function of time, Figure 5, and once again, we see an agreement between the values calculated through MaDDOSY and those obtained by conventional, offline, GPC analysis, in all cases with the GPC values being within the uncertainty of the MaDDOSY values.

**Figure 6:**
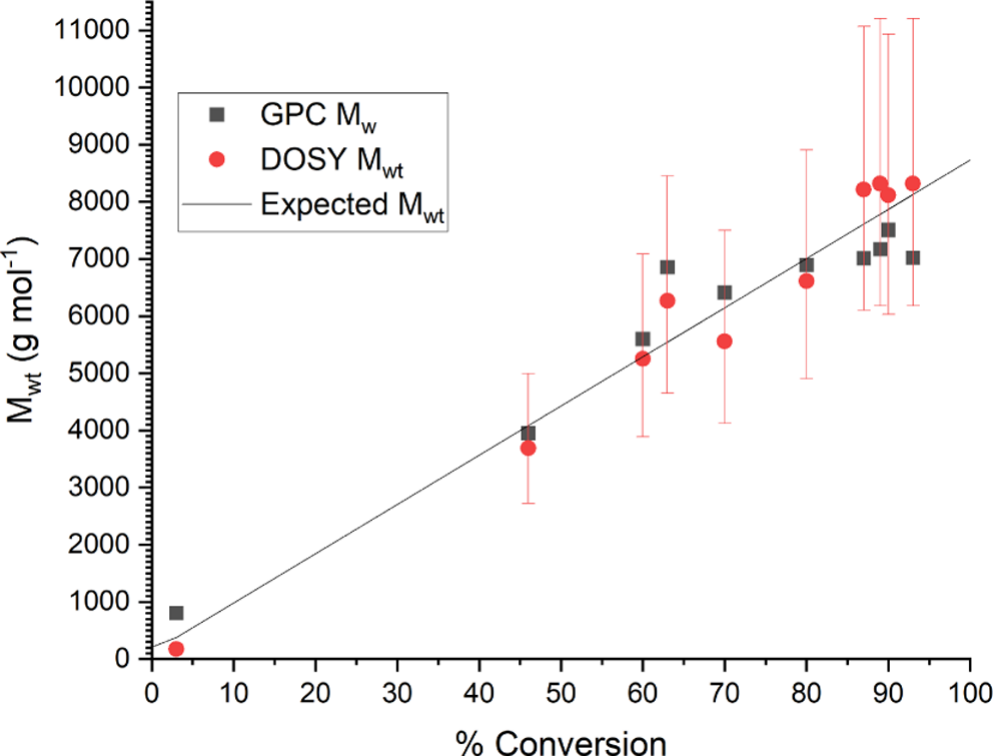
*M*_wt_ vs conversion for thermally initiated RAFT polymerization of methyl acrylate.

This agreement between the values obtained suggests that the viscosity correction provides a good approximation to account for the increased diffusion resulting from the higher temperature; thus, provided the viscosity at the experimental temperature is known, the system still performs appropriately. The *M*_wt_ also increased linearly with respect to monomer conversion; see Figure 6.

Thus, the experimentally derived molecular weight and the expected molecular weight have excellent agreement in this case, and unlike in the redox-initiated RAFT reaction, the reaction reaches a higher conversion of 90%, with the data remaining in agreement throughout the reaction. These two RAFT examples highlight the application of the technique to monitor reactions; however, the application should also be assessed against further mechanisms and monomers.

### Anionic Polymerization of Isoprene

**Figure 7:**
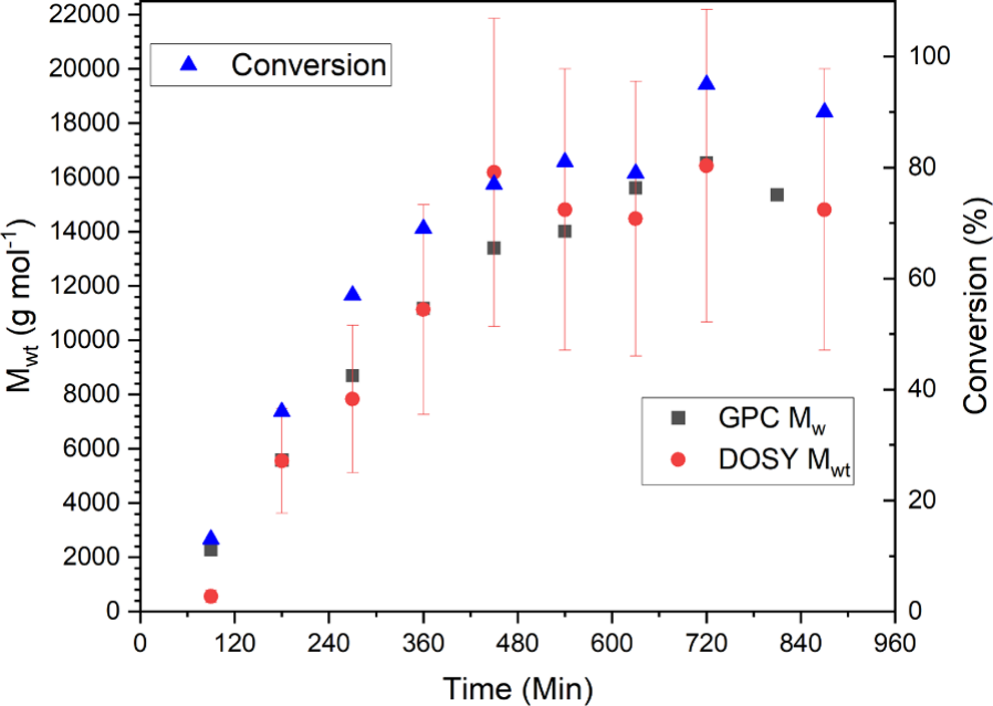
*M*_wt_ vs time for the anionic polymerization of isoprene.

The monitoring of anionic polymerizations in a laboratory presents different challenges to RAFT polymerization due to increased sensitivity to air and moisture. The benefit of the reaction setups described in Figures 1 and 2 is that they can be rendered airtight easily; therefore, molecular weight data can, in principle, be obtained without loss of any reaction mixture in the process. In this example, we used isoprene as the monomer, and during the initiation, an exotherm was observed, increasing the temperature and thus also the viscosity; this was taken into account when calculating the MaDDOSY molecular weights. Molecular weight increased as a function of time (Figure 7), reaching a larger final molecular weight than in other examples and good agreement between the GPC and MaDDOSY, thus demonstrating the applicability for polymers >10,000 g mol^–1^. One point of note is that the 95% confidence intervals are much larger at higher molecular weights; this is due to only 4 scans being performed at each gradient step. With more scans, these confidence intervals would become smaller, but in any case, the absolute values are very close to the GPC values, which themselves have a likely error of 10–20%.

**Figure 8:**
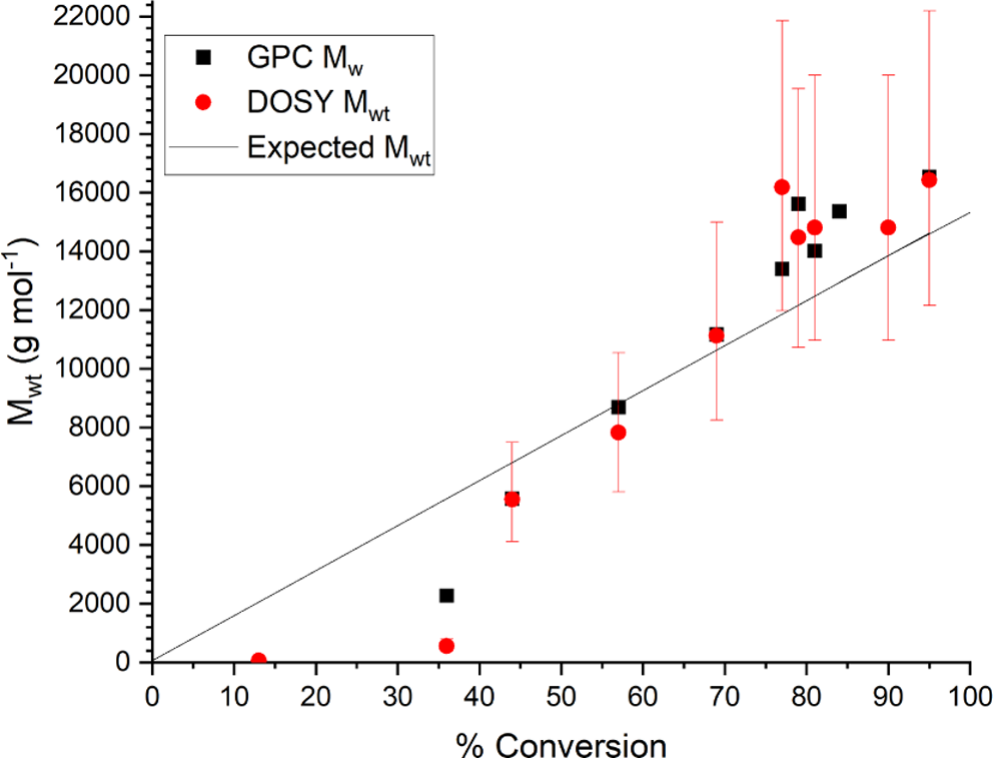
*M*_wt_ vs conversion for the anionic polymerization of isoprene.

As a living process, this polymerization should also display linearity in the number-average molecular weight vs conversion, and indeed, this is so in the case (Figure 8). Additionally, the high conversion of 90% suggests that the inert atmosphere of the reaction was not compromised as a result of the reaction monitoring setup, and the reaction proceeded as expected. The final dispersity was higher than would be hoped for anionic polymerization; however, this could be further optimized in subsequent experiments. It should be noted that in Figure 8, the values do not fall as expected. This is likely due to the relatively low molecular weights approaching the limits of the DOSY and GPC calibration.

Throughout these examples, it has been shown that the MaDDOSY reaction monitoring setup has excellent agreement with the result obtained by offline GPC. This gives us confidence about the versatility and robustness of the technique, provided care is taken when choosing the reaction conditions, as previously described. The most limiting of these comprises the comparatively lower concentrations, at or <10 wt %, required when compared to traditional bulk polymerizations.

### Photoinitiated Cu-RDRP Polymerization of Methyl Acrylate

Photoactivated polymerizations are of interest as they typically require considerably less energy than many other processes and can be easily controlled. To monitor a Cu-RDRP reaction, the reaction monitoring setup shown in Figure 2 was used, as the validity of the technique had already been demonstrated, showing good agreement between MaDDOSY and GPC.

**Figure 9:**
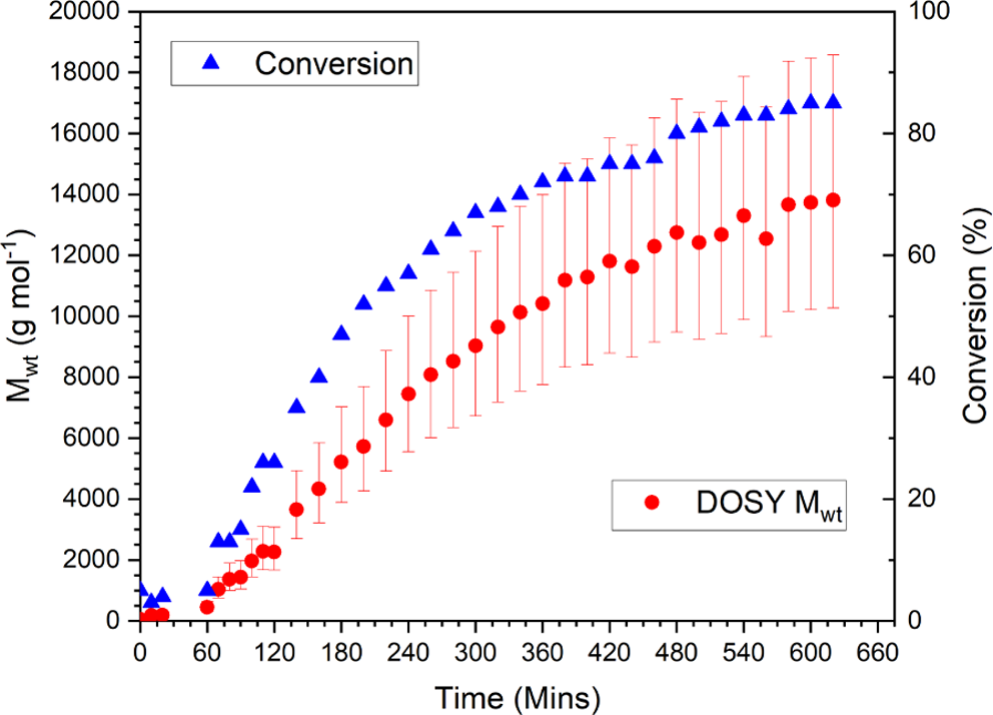
*M*_wt_ vs time for photoinitiated Cu-RDRP polymerization of methyl acrylate.

Figure 9 shows the increase in molecular weight as a function of time; over the course of the reaction, the change in molecular weight is clear and follows predictable growth.

**Figure 10:**
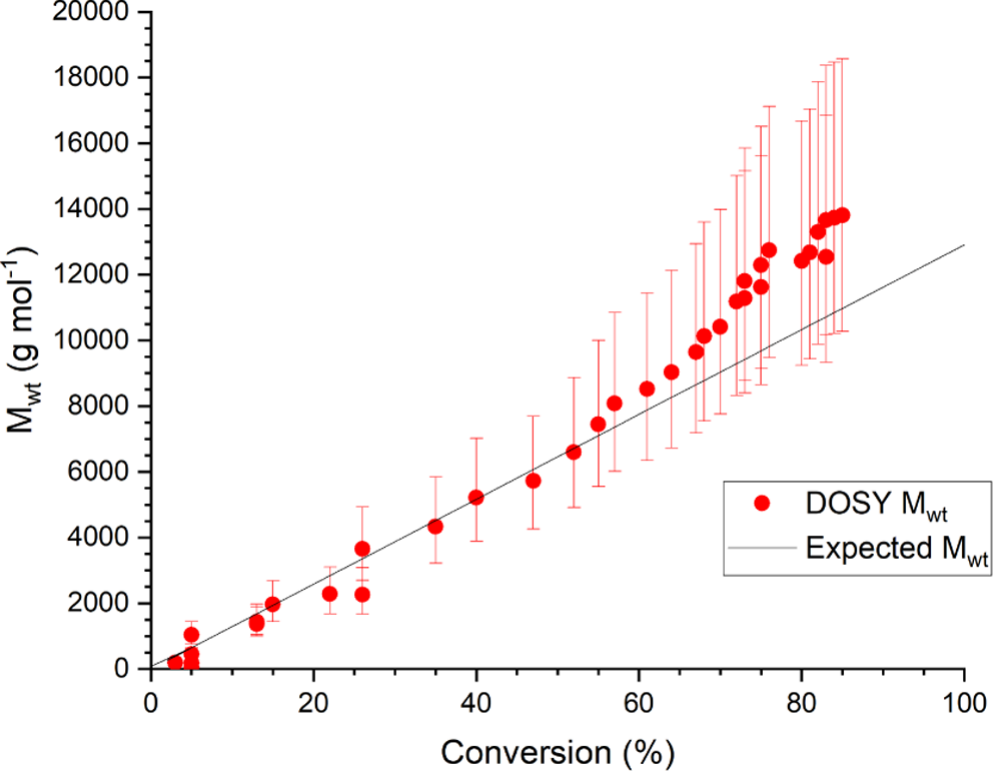
*M*_wt_ vs conversion for photoinitiated Cu-RDRP polymerization of methyl acrylate.

An additional change made with this setup was the reduction in the number of times the spectrometer was shimmed. In previous examples, shimming was performed after every DOSY measurement, whereas with this experiment, no shimming was performed, in order to examine the limits of equipment. This benchtop spectrometer has an external lock, meaning no deuterated solvents are required for locking or shimming of the magnet; however, this results in the magnetic field becoming inhomogeneous much faster than a high-field magnet. This leads to a deviation in the molecular weight from the expected molecular weight due to peak broadening, and therefore molecular weight calculation, at higher conversion (Figure 10). This notwithstanding, the expected molecular weight in all cases is within the predicted 95% confidence interval, which further demonstrates the robustness of MaDDOSY as a reaction monitoring technique. In addition, the peak broadening only occurred after approximately 5 h, suggesting that shimming is needed infrequently, potentially increasing the number of data points that can be acquired for a reaction.

## Conclusions

Building on our existing work of using mass determination diffusion ordered spectroscopy (MaDDOSY) as a tool for the calculation of polymer molecular weight, we have demonstrated its application as a useful and robust tool for reaction monitoring in real time. By using a standard flow cell and peristaltic pump, we have shown that a variety of different polymerization reactions can be monitored to provide close-to-real-time molecular weight information as the reaction proceeds.

RAFT polymerizations using two different methods of initiation at both room temperature and elevated temperature show that when accounting for the viscosity changes present as a result of temperature change, MaDDOSY can provide molecular weights similar to those obtained by conventional offline techniques; additionally, data can be collected much more frequently due to the faster analysis time with reporting in real time when performing a DOSY experiment compared to GPC analysis.

The application of the technique has also been demonstrated in a living anionic polymerization; this has shown that the analysis technique does not compromise the inert reaction conditions required for these polymerizations, making it an attractive option for analysis of complex systems.

A closed analysis system has also been demonstrated through the monitoring of a photoinitiated Cu-RDRP polymerization reaction, in which the reaction mixture is sampled, analyzed, and returned to the reaction mixture. In this setup, no reaction mixture is lost in the analysis, and the final product is unaffected by the analysis. This makes the technique an attractive option for analyzing small-scale reactions using potentially expensive reagents.

The limitations of the technique are discussed, which are primarily in relation to optimizing the reaction solvent and concentration prior to analysis. Additionally, the hardware limits the ability to perform the analysis in continuous flow; however, current work is being focused on resolving this. Future work should aim to test the system against more polymerization types, including step-growth and Ziegler–Natta catalyzed polymerizations, including potentially heterogeneous systems. Additionally, work will focus on using the output data to automate the variation of reaction conditions for fully automated polymer synthesis.

## Data Availability

Raw data is available upon request.

## Supporting Information Available

Representative spectra for each reaction, reaction schemes, and GPC chromatograms for each reaction (PDF)

## Abbreviations

AIBN: azobis(isobutyronitrile)
DMF: dimethylformamide
DMSO: dimethyl sulfoxide
DOSY: diffusion ordered spectroscopy
ESI: electrospray ionization
GPC: gel permeation chromatography
HPLC: high-performance liquid chromatography
MaDDOSY: mass determination diffusion ordered spectroscopy
MALDI: matrix-assisted laser desorption ionization
NMR: nuclear magnetic resonance
RAFT: reversible addition–fragmentation chain-transfer
RDRP: reversible-deactivation radical polymerization
ROP: ring-opening polymerization
SEC: size exclusion chromatography
