# Supplementary material for: Real-Time Determination of Molecular Weight: Use of MaDDOSY (Mass Determination Diffusion Ordered Spectroscopy) to Monitor the Progress of Polymerization Reactions
Source: ACS Polym Au. 2024 May 9;4(4):311–9. doi: 10.1021/acspolymersau.4c00020 (PMC11328330; doi:10.1021/acspolymersau.4c00020)
Supplement: Supplementary file 1 — lg4c00020_si_001.pdf [file lg4c00020_si_001.pdf]

Supplementary Information for: Real Time Determination of Molecular Weight: Use of MaDDOSY (Mass Determination Diffusion Ordered Spectroscopy) to Monitor the Progress of Polymerization Reactions

Owen Tooley[a], William Pointer[a], Rowan Radmall[a], Mia Hall[a][c], Thomas Swift[b], James Town[d], Cansu Aydogan[a], Tanja Junkers[c], Paul Wilson[a], Daniel Lester[d]* and David Haddleton[a][d]*

[a] Department of Chemistry, University of Warwick, Coventry, CV4 7AL, United Kingdom

[b] Department of Chemistry, University of Bradford, Bradford, West Yorkshire, BD7 1DP, United Kingdom

[c] School of Chemistry, Monash University, 17 Rainforest Walk, Clayton VIC, 3800 Australia

[d] Polymer Characterization RTP, University of Warwick, Coventry, CV4 7AL, United Kingdom

Diffusion Ordered Spectroscopy, NMR, Polymerization, Reaction Monitoring

EXPERIMENTAL

REDOX INITIATED RAFT POLYMERIZATION OF METHYL ACRYLATE

The scheme for the redox-initiated RAFT polymerization of Methyl Acrylate is shown in scheme 1, with a representative NMR spectrum shown in figure 1. Initial and final DOSY spectra are shown in figure 2. The structure of the RAFT agent, 2-(((butylthio)carbonothioyl)thio)propanoic acid (PABTC) is shown in structure 1.

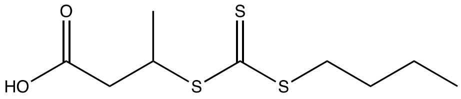

Structure 1- RAFT Agent PABTC

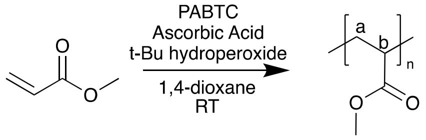

Scheme 1-Redox Initiated Polymerisation of Methyl Acrylate

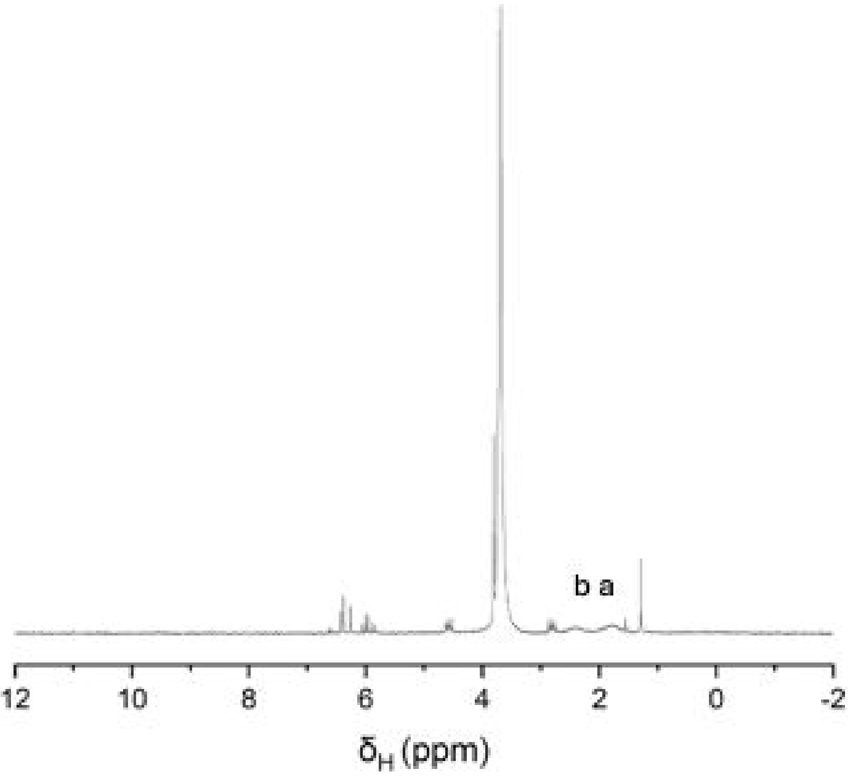

Figure 1 - ^{1}H NMR (80 MHz) spectrum of Redox Initiated RAFT polymerization of Poly(Methyl Acrylate)

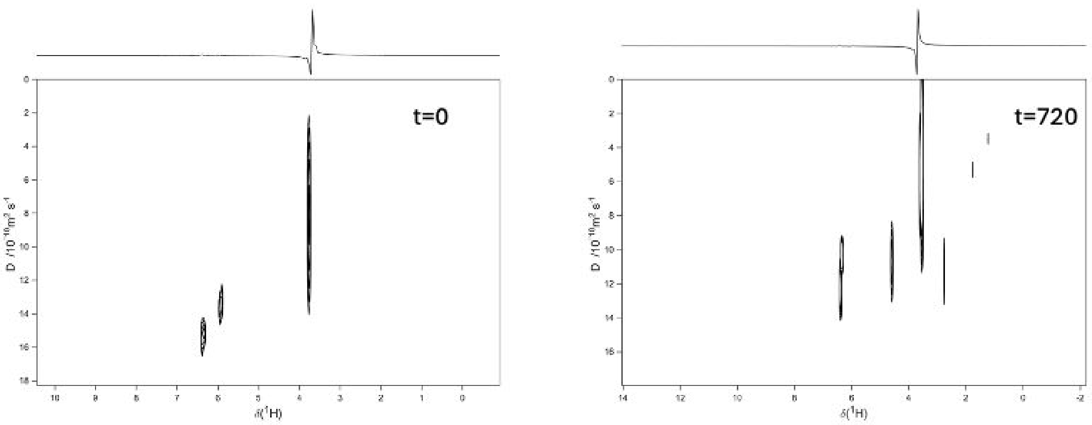

Figure 2 - t=0 and t=720 DOSY spectra for redox initiated RAFT polymerization of Methyl Acrylate

The GPC traces, collected on the differential refractive index (DRI) detector using a PMMA calibration, are shown in figure 3. The trace for t=0 is included, however is suboptimal, due to elution occurring near the solvent system peak.

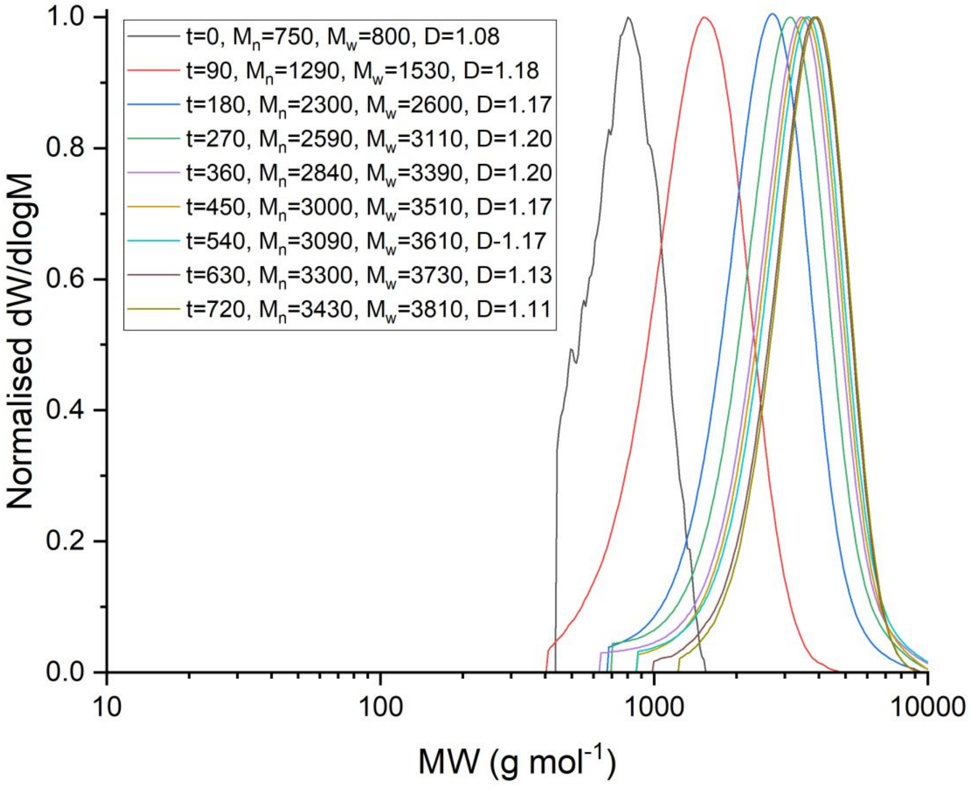

Figure 3 - GPC traces for redox initiated polymerization of Methyl Acrylate

THERMALLY INITIATED RAFT POLYMERIZATION OF METHYL ACRYLATE

The scheme for the thermally RAFT polymerization of Methyl Acrylate is shown in scheme 2, with a representative NMR spectrum shown in figure 4. Initial and final DOSY spectra are shown in figure 5.

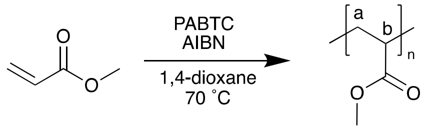

Scheme 2-Thermally initiated RAFT polymerization of Methyl Acrylate

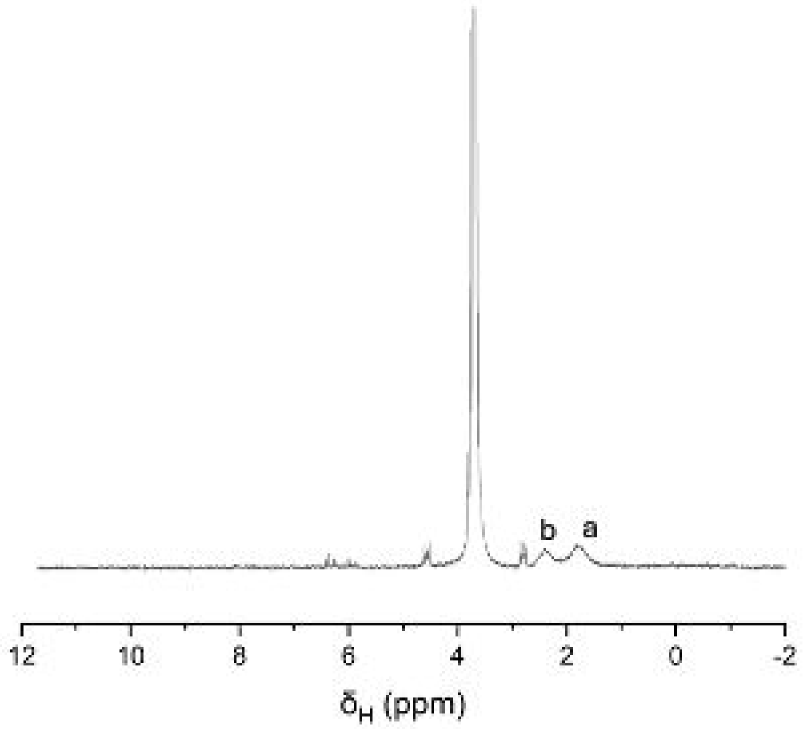

Figure 4 - ^{1}H NMR (80 MHz) spectrum of thermally Initiated RAFT polymerization of Poly(Methyl Acrylate)

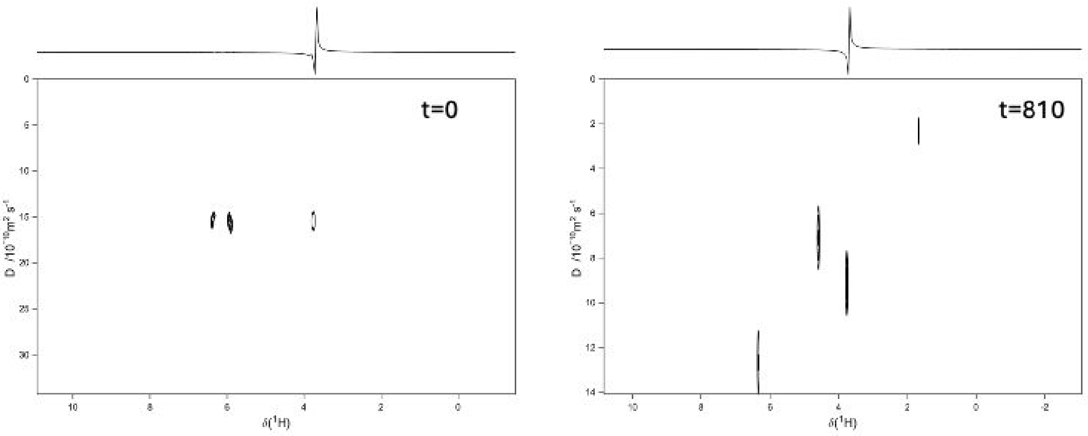

Figure 5 - t=0 and t=810 DOSY spectra for thermally initiated RAFT polymerization of Methyl Acrylate

The GPC traces, collected on the differential refractive index (DRI) detector using a PMMA calibration, are shown in figure 6. The trace for t=0 is included, however is suboptimal, due to elution occurring near the solvent system peak.

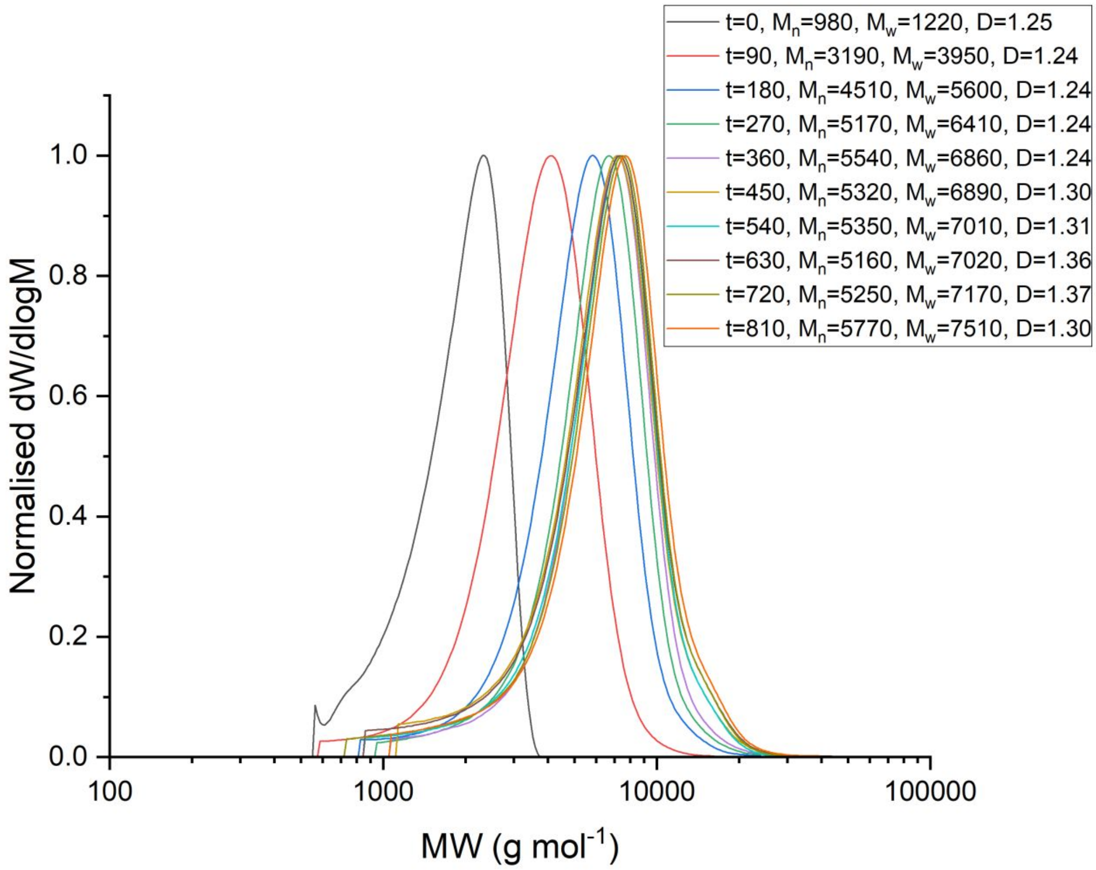

Figure 6 - GPC traces for thermally initiated polymerization of Methyl Acrylate

As discussed in the main text, an approximation of the viscosity had to be used in this experiment, due to the elevated temperature associated with the polymerization conditions. The viscosity of course makes an incredibly large difference to the predicted molecular weight, as shown in figure 7 for the diffusion constant measured in the t=630 sample. When comparing to the GPC results, we can see that there is a cooling effect in the tubing of approximately 5 °C .

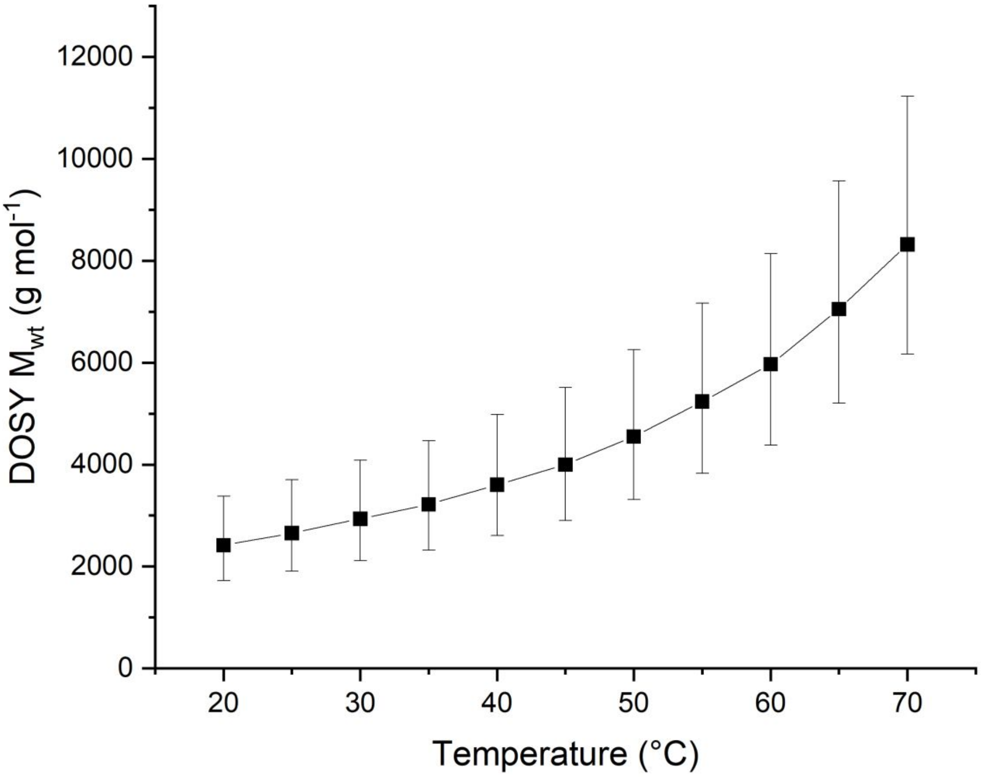

Figure 7 - Temperature vs Predicted M_{wt} for t=630 sample

ANIONIC POLYMERIZATION OF ISOPRENE

The scheme for the anionic polymerization of isoprene is shown in scheme 3, with a representative NMR spectrum shown in figure 8. Initial and final DOSY spectra are shown in figure 9.

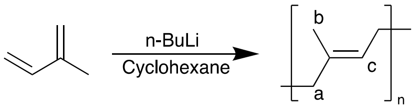

Scheme 3 - Anionic polymerization of Isoprene

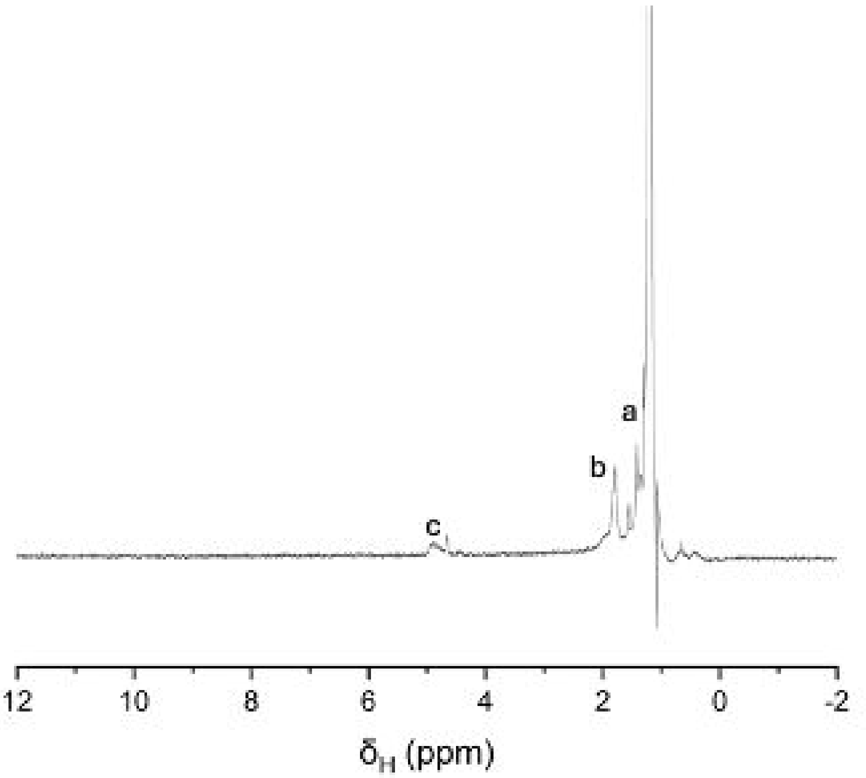

Figure 8 - ^1H NMR (80 MHz) spectrum of anionic polymerization of Isoprene.

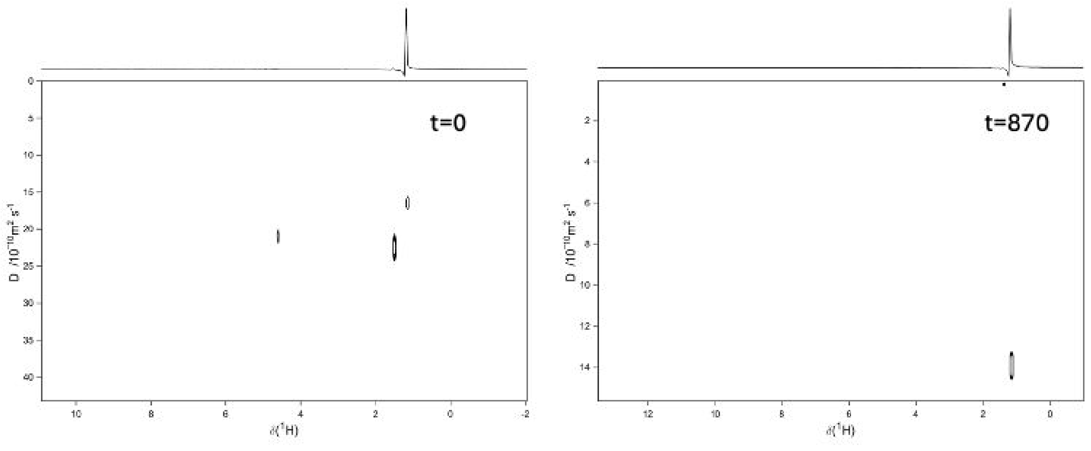

Figure 9 - t=0 and t=870 DOSY spectra for anionic polymerization of Isoprene.

The GPC traces, collected on the differential refractive index (DRI) detector using a PMMA calibration, are shown in figure 10. The trace for t=0 is omitted due to coelution with the solvent system peak. The trace for t=90 is suboptimal due to elution of the lower molecular weight species near the solvent system peak. The baselines in these chromatograms were noisy, and so baseline correction here was not ideal, however the M_w values needed for comparison with DOSY are not noticeably affected.

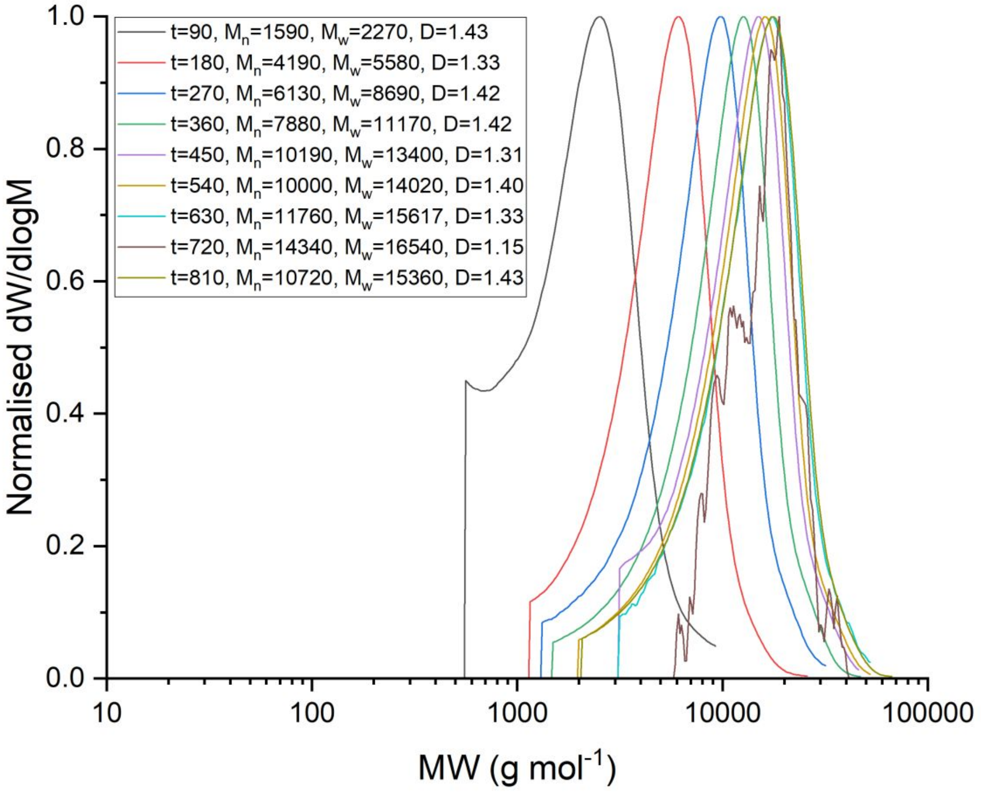

Figure 10 - GPC traces of anionic polymerization of Isoprene

PHOTO-INITIATED CU-RDRP POLYMERIZATION OF METHYL ACRYLATE

The scheme for the photo-initiated Cu-RDRP polymerization of methyl acrylate is shown in scheme 4, with a representative NMR spectrum shown in figure 11. Initial and final DOSY spectra are shown in figure 12.

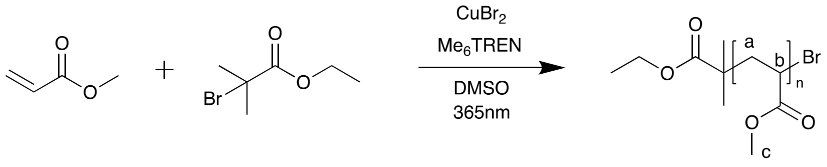

Scheme 4 - Photo-initiated Cu-RDRP polymerization of Methyl Acrylate

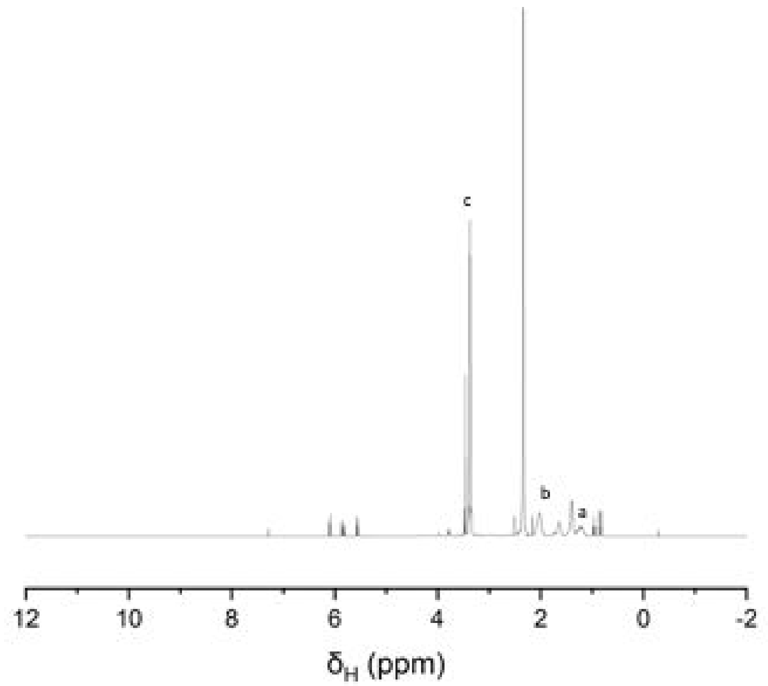

Figure 11 - ^{1}H NMR (80 MHz) spectrum of photo-initiated Cu-RDRP polymerization of Methyl Acrylate

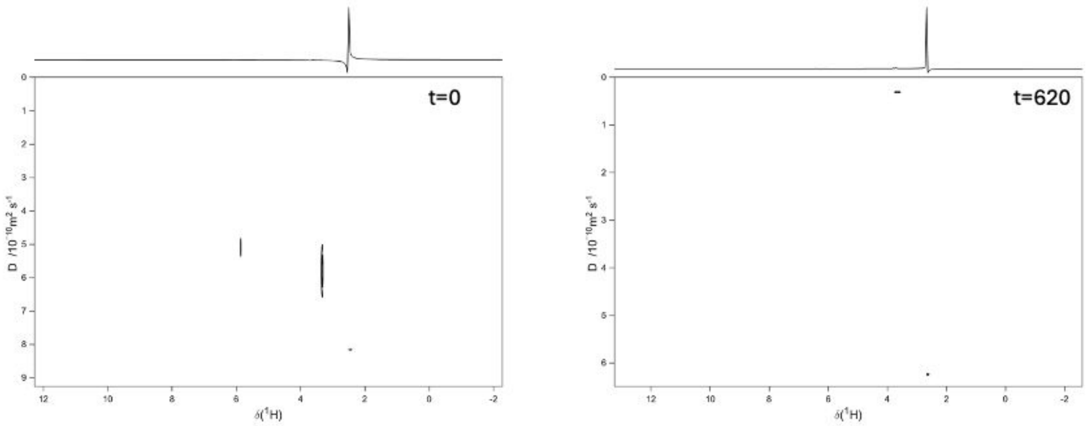

Figure 12 - t=0 and t=620 DOSY spectra of the photo-initiated Cu-RDRP polymerization of Methyl Acrylate

As GPC chromatograms were not collected throughout this reaction, a chromatogram of the final product was collected on the differential refractive index (DRI) detector using a PMMA calibration and is shown in figure 13.

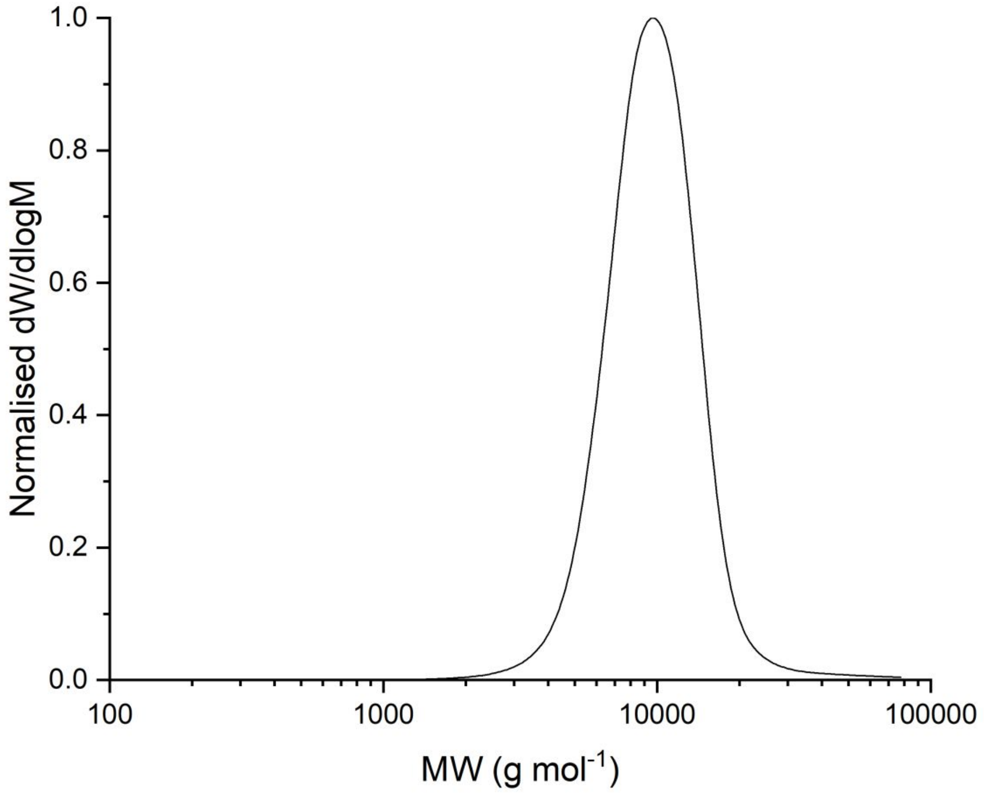

Figure 13 - Final product GPC trace from photo-initiated Cu-RDRP polymerization of Methyl Acrylate

DOSY NMR SPECTRA PROCESSING

When DOSY spectra were processed directly in Spinsolve version 2.3.6, diffusion constants are calculated through a Stejskal-Tanner plot. In this case, the area of a given region (which is a polymer peak) is plotted against the gradient strength, and the gradient of the resultant line provides the diffusion constant. For reference, a stacked spectrum plot, and resultant Stejskal-Tanner plot for a 100, 000 g mol^{-1} sample of polyisoprene are shown in figures 14 and 15. When diffusion constants were calculated using the GNAT, version 1.3.2, developed by the Manchester NMR Methodology Group, a pseudo-2D plot is generated, with diffusion constants calculated for all peaks shown on the y-axis, against chemical shift on the x-axis. Here, Stejskal-Tanner plots are also used, but are generated automatically. An example of this approach for the same sample is shown in figure 16.

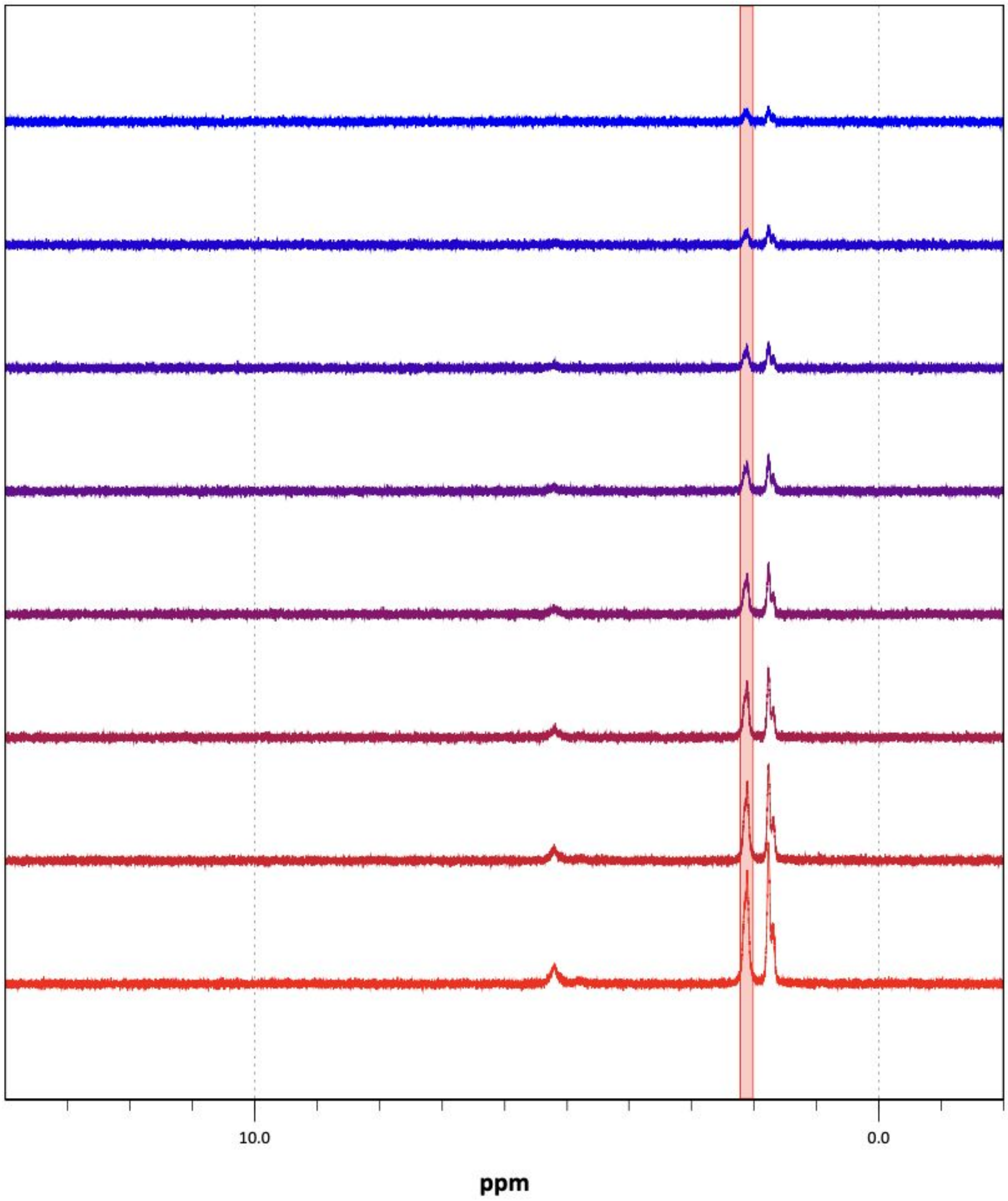

Figure 14 - Stacked ^{1}H NMR spectra at increasing gradient strengths shown for 100, 000 g mol^{-1} sample of polyisoprene, output from Spinsolve version 2.3.6

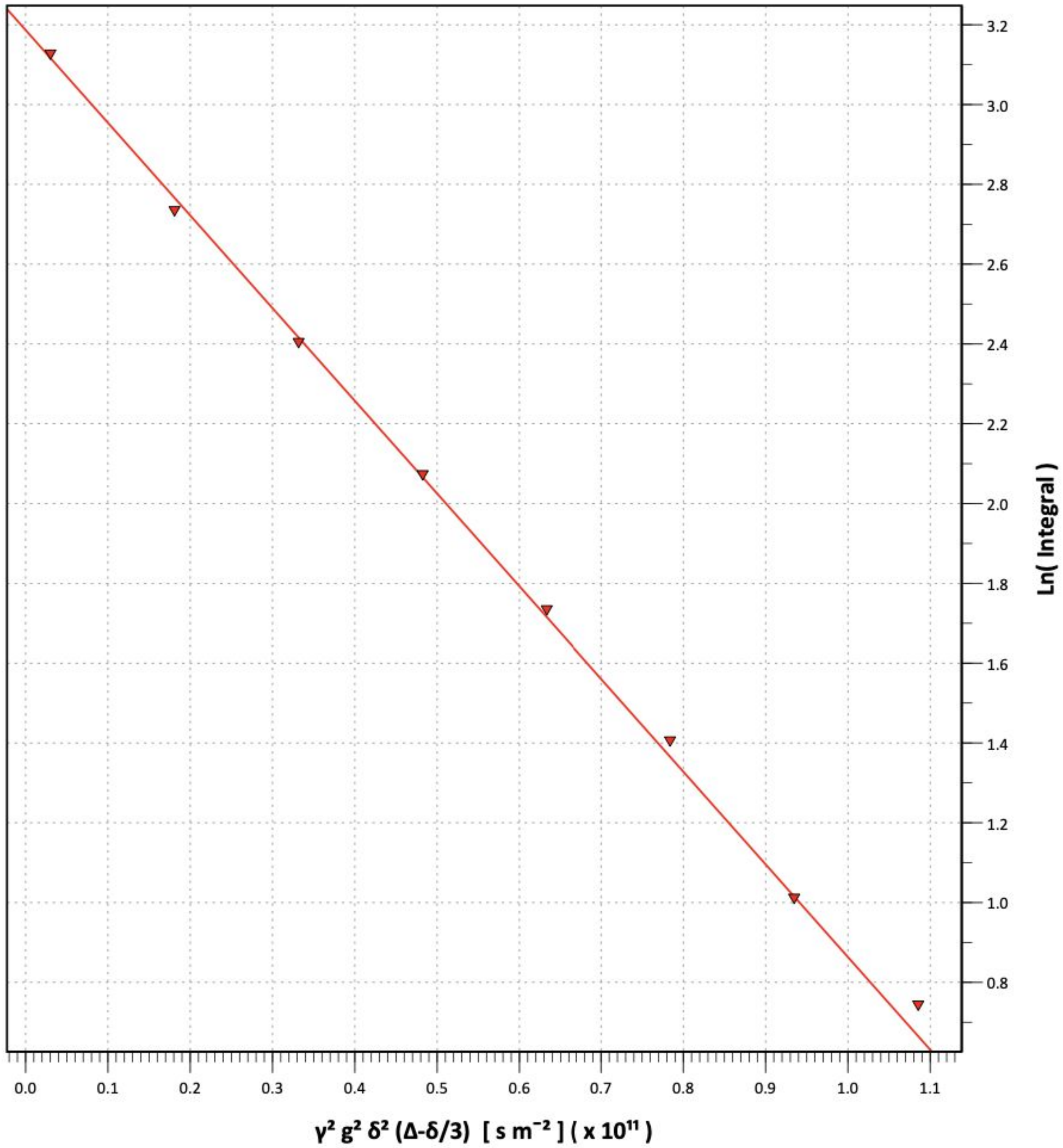

Figure 15 – Resultant Stejskal-Tanner plot from100, 000 g mol^{-1} sample of polyisopreneoutput from Spinsolve version 2.3.6

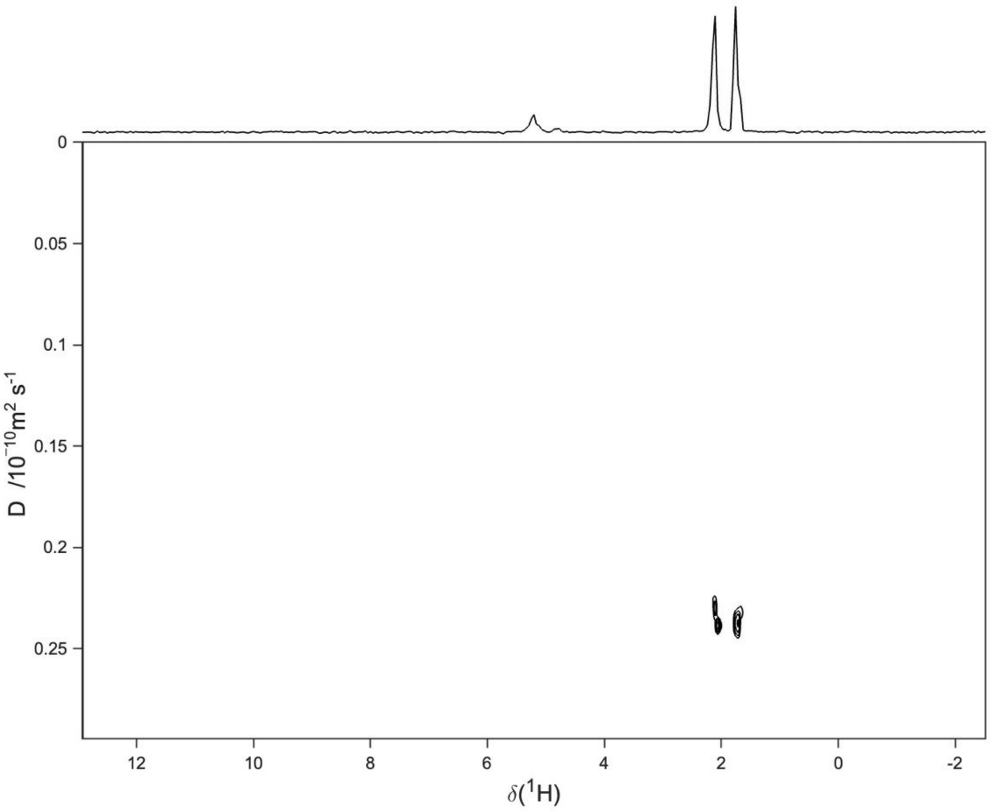

Figure 16- Pseudo-2D DOSY plot for a sample of 100, 000 g mol^{-1} polyisoprene, output from GNAT version 1.3.2
